# Novel Insights on Establishing Machine Learning-Based Stroke Prediction Models Among Hypertensive Adults

**DOI:** 10.3389/fcvm.2022.901240

**Published:** 2022-05-06

**Authors:** Xiao Huang, Tianyu Cao, Liangziqian Chen, Junpei Li, Ziheng Tan, Benjamin Xu, Richard Xu, Yun Song, Ziyi Zhou, Zhuo Wang, Yaping Wei, Yan Zhang, Jianping Li, Yong Huo, Xianhui Qin, Yanqing Wu, Xiaobin Wang, Hong Wang, Xiaoshu Cheng, Xiping Xu, Lishun Liu

**Affiliations:** ^1^Department of Cardiology, The Second Affiliated Hospital of Nanchang University, Nanchang, China; ^2^Biological Anthropology, University of California, Santa Barbara, Santa Barbara, CA, United States; ^3^Department of Data Management, Shenzhen Evergreen Medical Institute, Shenzhen, China; ^4^Department of Epidemiology, Harvard T.H. Chan School of Public Health, Boston, MA, United States; ^5^Department of Biostatistics, Johns Hopkins Bloomberg School of Public Health, Baltimore, MD, United States; ^6^Institute of Biomedicine, Anhui Medical University, Hefei, China; ^7^Department of Biomedical Engineering, Graduate School at Shenzhen, Tsinghua University, Shenzhen, China; ^8^Key Laboratory of Precision Nutrition and Food Quality, Ministry of Education, Department of Nutrition and Health, College of Food Sciences and Nutritional Engineering, China Agricultural University, Beijing, China; ^9^Department of Cardiology, Peking University First Hospital, Beijing, China; ^10^National Clinical Research Study Center for Kidney Disease, The State Key Laboratory for Organ Failure Research, Renal Division, Nanfang Hospital, Southern Medical University, Guangzhou, China; ^11^Department of Population, Family and Reproductive Health, Johns Hopkins University Bloomberg School of Public Health, Baltimore, MD, United States; ^12^Department of Cardiovascular Science, Temple University Lewis Katz School of Medicine, Philadelphia, PA, United States

**Keywords:** machine learning, risk assessment, stroke, primary prevention, XGBoost

## Abstract

**Background:**

Stroke is a major global health burden, and risk prediction is essential for the primary prevention of stroke. However, uncertainty remains about the optimal prediction model for analyzing stroke risk. In this study, we aim to determine the most effective stroke prediction method in a Chinese hypertensive population using machine learning and establish a general methodological pipeline for future analysis.

**Methods:**

The training set included 70% of data (*n* = 14,491) from the China Stroke Primary Prevention Trial (CSPPT). Internal validation was processed with the rest 30% of CSPPT data (*n* = 6,211), and external validation was conducted using a nested case–control (NCC) dataset (*n* = 2,568). The primary outcome was the first stroke. Four received analysis methods were processed and compared: logistic regression (LR), stepwise logistic regression (SLR), extreme gradient boosting (XGBoost), and random forest (RF). Population characteristic data with inclusion and exclusion of laboratory variables were separately analyzed. Accuracy, sensitivity, specificity, kappa, and area under receiver operating characteristic curves (AUCs) were used to make model assessments with AUCs the top concern. Data balancing techniques, including random under-sampling (RUS) and synthetic minority over-sampling technique (SMOTE), were applied to process this unbalanced training set.

**Results:**

The best model performance was observed in RUS-applied RF model with laboratory variables. Compared with null models (sensitivity = 0, specificity = 100, and mean AUCs = 0.643), data balancing techniques improved overall performance with RUS, demonstrating a more satisfactory effect in the current study (RUS: sensitivity = 63.9; specificity = 53.7; and mean AUCs = 0.624. Adding laboratory variables improved the performance of analysis methods. All results were reconfirmed in validation sets. The top 10 important variables were determined by the analysis method with the best performance.

**Conclusion:**

Among the tested methods, the most effective stroke prediction model in targeted population is RUS-applied RF. From the insights, the current study revealed, we provided general frameworks for building machine learning-based prediction models.

## Introduction

Stroke is the leading cause of death in China (1). Stroke management and prevention methods are urgently needed, especially in Chinese rural areas, which bear the heaviest stroke burden (2). Primary prevention of stroke is the top priority, and more than 85% of strokes are preventable (3). The key is to develop effective stroke prediction methods and identify important stroke risk factors.

Machine learning has been validated as an effective data analyzing method and has seen growing usage in epidemiological studies and the field of medicine (4, 5). Its strengths include ease of analysis and the ability to simultaneously consider a huge number of variables and capture complex interactions between variables. For these reasons, machine learning has garnered favor as an analysis method in some research over traditional regression models (6, 7). However, some important methodological questions remain unanswered. Across different studies, the optimal model often differs, and the appropriate balance of variables to include in the model differs as well.

The current study aimed to explore the optimal stroke prediction method by using two classic logistic regression methods and two currently admitted machine learning models. The main data were obtained from a large-scale RCT study and a nested case–control study, which shared similar data characteristics. The targeted population is Chinese rural area hypertensive adults without a prior history of stroke. With the large sample size and thorough data processing process, we also try to establish general framework for future analysis that builds prediction method using machine learning.

## Methods

### Study Population

Two datasets with similar baseline characteristics investigated by the same team were selected and analyzed in our study: the China Stroke Primary Prevention Trial (CSPPT) dataset and the nested case–control (NCC) dataset which is a subset from the H-type Hypertension and Stroke Prevention and Control Project (HSPCP).

In brief, CSPPT is a multicenter, double-blinded, randomized control trial conducted in 32 communities in Jiangsu (20 communities) and Anhui (12 communities) provinces from May 19, 2008, to August 24, 2013, in China. This study has been thoroughly described before (8). Eligible participants of the CSPPT study included hypertensive men and women aged 45–75 years, with hypertension defined as seated resting SBP (systolic blood pressure) of 140 mmHg and higher; or DBP (diastolic blood pressure) of 90 mmHg and higher during screening and follow-up visits; or using antihypertensive medication. HSPCP, which has also been thoroughly described previously (9), is an ongoing community-based, multicenter, non-interventional, prospective, observational, real-world registry study. Eligible subjects for the HSPCP study were men and women aged 18 years or older with essential hypertension, defined as seated resting SBP more than or equal to 140 mmHg or DBP more than or equal to 90 at baseline. Both studies were approved by the Ethics Committee of the Institute of Biomedicine, Anhui Medical University, Hefei, China, and all participants from both studies provided written informed consent.

### Predictors and Data Processing

Baseline data, including demographic characteristics, traditional risk factors, medication usage, questionnaire information, physical examinations, and laboratory tests, were collected by trained employees. After careful selection, important variables that presented the most in both the training and validating datasets (intersection) were entered into the final analysis, such as blood pressure, laboratory data, cardiovascular risk factors, and medication use. Furthermore, to explore the additive value on model performance, laboratory variables were excluded in one subgroup analysis and included in another (with or without laboratory test data).

### Outcome Assessment

The primary outcome was new nonfatal and fatal stroke (ischemic or hemorrhagic) occurring between baseline and follow-up (a median of 4.2 years). Silent stroke and subarachnoid hemorrhage were excluded. All source data of suspected stroke cases, including imaging data, event reports, and medical records, were collected and further validated by the event adjudication committee (8).

### Analysis Methods Tested

Four data analysis methods were tested which included two logistic regression methods: logistic regression (LR) and stepwise logistic regression (SLR) and two machine learning methods: random forest (RF) and XGBoost.

Two logistic regression analysis methods:

Logistic regression (LR) analyzed the relationship between multiple independent influencing factors and a categorical or binary outcome. By controlling confounding influencing factors and seizing important factors, logistic regression is able to make probabilistic predictions toward the selected outcome (10).

Stepwise logistic regression (SLR) is a semi-automated analysis method that continuously adds or removes variables from the model at each step. It is useful in a database with a large number of independent variables (11).

Two machine learning methods:

Extreme gradient boosting (XGBoost) can generate a collection of classification trees and assign each variable with a predictive risk score (12), which is an improved algorithm based on the GDBT (gradient boosting decision tree). XGBoost performs the second-order Taylor expansion of the cost function and adds a regularization item to achieve better performance. It adds predictions from weak regression trees sequentially to maximize model performance and minimize model complexity while avoiding over-fitting. XGBoost has become one of the most accepted models for risk identification and event prediction.

Random forest (RF) is a widely used learning method that produces an ensemble of decision trees with random variables as branches. By using the majority principle from all of the trees and branches, RF is able to make predictions with high accuracy with less over-fitting and strong anti-noise ability (13). It is a combined classifier algorithm based on the cart decision tree. Following the principle that a minority is subordinate to the majority, RF votes decision trees in the forest and the category with higher votes can be determined.

Two data balancing techniques:

The stroke-to-non-stroke ratio was approximately 1:31, which suggested an imbalance. Random under-sampling (RUS) and synthetic minority over-sampling technique (SMOTE) were used as data balancing techniques in the training dataset. RUS is a commonly used data balancing technique that randomly removes samples from the majority dataset until it reaches a size equivalent to the minority dataset (14). The synthetic minority over-sampling technique (SMOTE) randomly generates synthetic data to increase the minority instances based on similarities between the nearest data neighbors (15).

### Internal and External Validation

**Figure 1:**
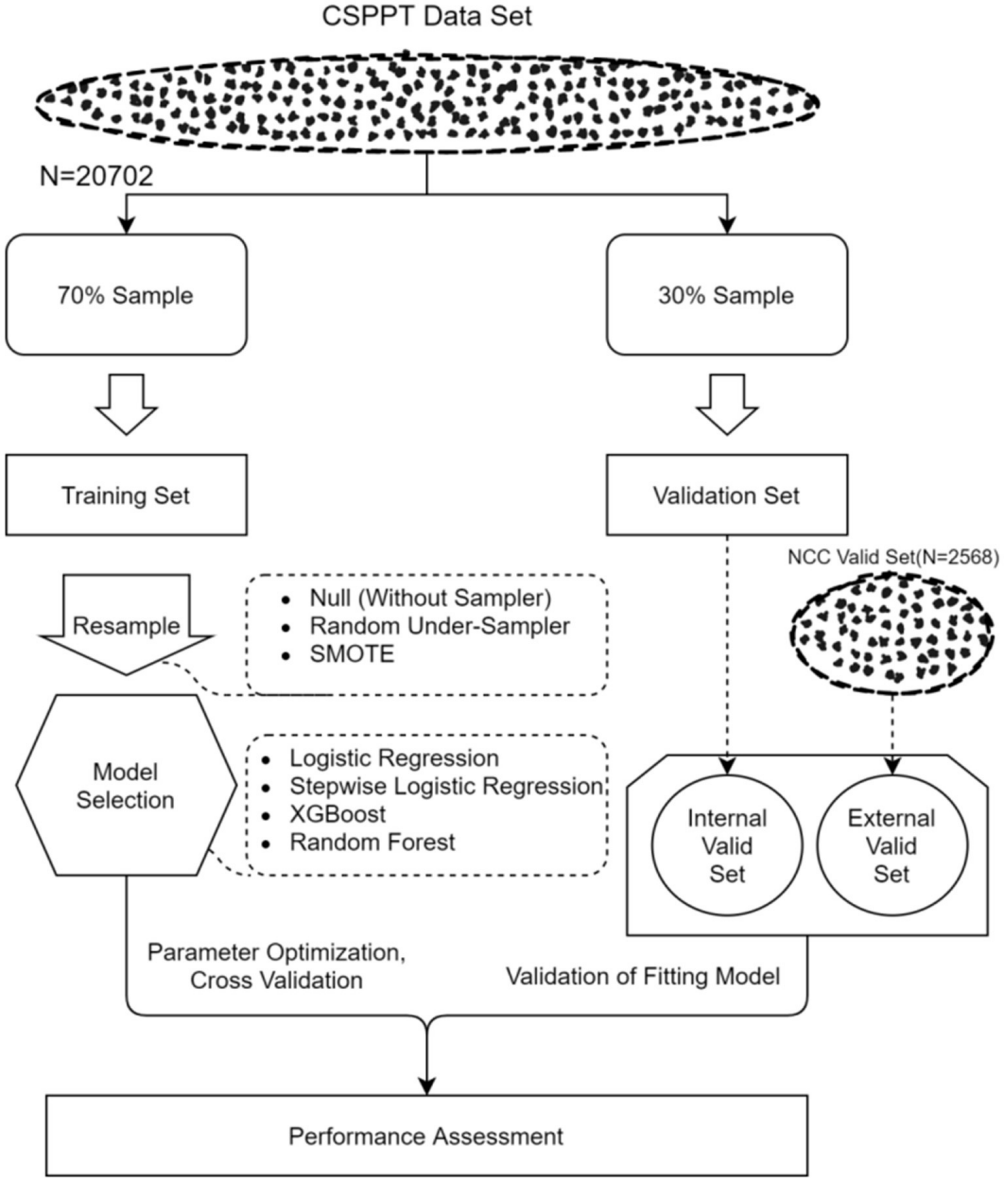
Analysis flow for the development and evaluation of models.

As mentioned in the study population section (Figure 1), the total CSPPT dataset was divided into a training set (70%, *n* = 14,491) an internal validation set (30%, *n* = 6,211); and the NCC dataset, which possesses similar data characteristics with the CSPPT set, was treated as external validation. In brief, a stroke predictive model was trained on the training set. Then, data from the internal and external validation sets were used to treat the aforementioned predictive model to get predictive results. These predictive results were compared with the actual observed results, respectively, from which the processed AUCs can be obtained. At last, AUCs for both validation sets were compared to assess the accuracy and universality of the trained predictive model. In addition, 10-fold cross-validation was applied to each analysis model for derivation and validation. In computational-heavy analyses, a 10-fold CV can improve the accuracy and efficiency of the prediction model by reducing the MSE, bias, and variance (16). Moreover, a 10-fold CV can avoid type III errors (arbitrarily split data suggested testing hypotheses). Ten-fold CV randomly divides data into 10 equal folds, and then, each fold in turn is used as the validation set, while the nine other folds are used as the training set.

### Statistical Analysis

Continuous variables are presented as mean with standard deviation (SD, normal distribution) and as medians with inter-quartile range (IQR, skewed distribution). Categorical variables are presented as percentages. The *t*-test, Wilcoxon rank-sum test, and the Chi-square test were used for statistical comparison between stroke and non-stroke populations. Sensitivity, specificity, accuracy, kappa, and areas under the receiver operating characteristic curve (AUCs) were used to make the model assessment with AUCs the top concerns for model performance evaluation. Data balancing techniques, including RUS and SMOTE, were applied to process the imbalanced dataset (stroke-to-non-stroke incidence was 1:31) meanwhile separately compared with each other and the null model (regression coefficients equal to 0). Box plots were generated to explore the efficacy of the inclusion of laboratory data. Receiver operating characteristics curves were generated to examine and compare the performance of four analysis methods in both the CSPPT and NCC datasets.

Two-tailed *P* < 0.05 was considered significant in all analyses. All statistical analyses were performed using R software, version 3.5.2 (http://www.R-project.org/, accessed 20 December 2018).

## Results

### Baseline Characteristics

CSPPT dataset (training and internal validation dataset): A total of 20,702 rural Chinese hypertensive participants without a prior history of stroke at baseline were included. In the targeted population, 41% were male (*n* = 8,497) and had a mean age of 60.0 (SD: 7.5) and a mean SBP of 165.8 (SD 18.3) mmHg at baseline. During a median follow-up period of 4.5 years, 637 new stroke cases occurred (3.1% of the total population). The stroke incidence rate was approximately 3,225.81/100,000. Statistical significances (*P* < 0.05) were observed regarding age, BMI, DBP, SBP, ALB, AST, GGT, CHOL, GLU, CREA, sex, diabetes, smoking, fruit intake, and antihypertensive drugs usage (*P* < 0.05).

NCC (external validation set): A total of 2,568 hypertensive patients with a mean age of 70.6 (SD 8.2), 51.1 % being male (*n* = 1,311), and having a 153.1 (SD 22.8) mmHg mean SBP level were entered into the final analysis. The stroke cases-to-non-stroke cases ratio was 1:1 with number equal to 1,284. With identical variables to the CSPPT dataset, differences were observed in hip, BMI, DBP, SBP, pulse, calcium, triglycerides, glucose, diabetes, antihypertensive drugs usage, and hypoglycemic drugs usage (*P* < 0.05; Table 1).

**Table 1:** Baseline and follow-up characteristics of the study participants.

**Dataset**	**CSPPT**				**NCC validation**			
	**Total**	**Stroke**	**No stroke**	***P-*Value**	**Total**	**Stroke**	**No stroke**	***P-*Value**
*N*	20,702	637	20,065		2,568	1,284	1,284	
Sex				0.001				0.97
Male	8,497 (41.0)	302 (47.4)	8,195 (40.8)		1,311 (51.1)	656 (51.1)	655 (51.0)	
Female	12,205 (59.0)	335 (52.6)	11,870 (59.2)		1,257 (48.9)	628 (48.9)	629 (49.0)	
Age, year	60.0 (7.5)	62.2 (7.3)	59.9 (7.5)	<0.001	70.6 (8.2)	70.6 (8.2)	70.6 (8.2)	0.99
Hip, cm	94.6 (6.9)	94.7 (7.2)	94.6 (6.9)	0.73	98.1 (8.5)	98.7 (8.4)	97.5 (8.5)	0.001
BMI, kg/m^2^	24.8 (3.5)	25.1 (3.6)	24.8 (3.5)	0.04	26.3 (4.1)	26.6 (4.3)	26.0 (3.7)	<0.001
DBP, mmHg	93.8 (10.9)	95.8 (11.0)	93.8 (10.8)	<0.001	85.5 (12.3)	87.4 (12.8)	83.6 (11.5)	<0.001
SBP, mmHg	165.8 (18.3)	173.1 (18.5)	165.5 (18.3)	<0.001	153.1 (22.8)	157.0 (23.4)	149.2 (21.4)	<0.001
Pulse, BMP	72.9 (8.7)	72.9 (8.7)	72.9 (8.7)	0.93	74.5 (12.7)	75.2 (12.8)	73.7 (12.5)	0.006
Laboratory data								
Albumin, mmol/L	48.6 (4.2)	48.3 (4.2)	48.6 (4.2)	0.02	47.0 (3.2)	47.0 (3.1)	47.1 (3.2)	0.21
AST, mmol/L	24.5 (6.8)	23.6 (6.8)	24.5 (6.8)	0.001	21.9 (14.4)	21.5 (8.9)	22.4 (18.4)	0.11
γ-GT, mmol/L	21.9 (9.6)	23.3 (10.2)	21.9 (9.5)	<0.001	27.0 (27.9)	27.4 (25.7)	26.6 (29.9)	0.49
TC, mmol/L	5.5 (1.1)	5.6 (1.0)	5.5 (1.1)	<0.001	5.8 (1.2)	5.8 (1.2)	5.8 (1.2)	0.56
Calcium, mmol/L	2.6 (0.2)	2.6 (0.2)	2.6 (0.2)	0.30	2.3 (0.2)	2.4 (0.2)	2.3 (0.2)	0.04
Triglycerides, mmol/L	1.5 (0.6)	1.5 (0.6)	1.5 (0.6)	0.81	1.4 (0.9)	1.5 (0.9)	1.3 (0.8)	<0.001
Glucose, mmol/L	5.6 (0.8)	5.7 (0.9)	5.6 (0.8)	<0.001	6.2 (2.3)	6.5 (2.5)	6.0 (2.0)	<0.001
Creatinine, mmol/L	64.6 (13.2)	66.1 (13.8)	64.6 (13.2)	0.005	60.2 (27.8)	61.2 (27.4)	59.2 (28.2)	0.08
Cardiovascular risk factors								
Diabetes				<0.001				<0.001
No	18,414 (88.9)	521 (81.8)	17,893 (89.2)		2,133 (83.1)	1,019 (79.4)	1,114 (86.8)	
Yes	2,288 (11.1)	116 (18.2)	2,172 (10.8)		435 (16.9)	265 (20.6)	170 (13.2)	
Smoking				<0.001				0.06
Never	14,263 (68.9)	387 (60.8)	13,876 (69.2)		1,745 (68.0)	856 (66.7)	889 (69.2)	
Former	1,570 (7.6)	62 (9.7)	1,508 (7.5)		256 (10.0)	122 (9.5)	134 (10.4)	
Current	4,869 (23.5)	188 (29.5)	4,681 (23.3)		567 (22.1)	306 (23.8)	261 (20.3)	
Alcohol drinking				0.08				0.66
Never	14,283 (69.0)	415 (65.1)	13,868 (69.1)		1,850 (72.0)	923 (71.9)	927 (72.2)	
Former	1,459 (7.0)	57 (8.9)	1,402 (7.0)		95 (3.7)	61 (4.8)	34 (2.6)	
Current	4,960 (24.0)	165 (25.9)	4,795 (23.9)		623 (24.3)	300 (23.4)	323 (25.2)	
Living standard				0.09				0.43
Good	2,476 (12.0)	72 (11.3)	2,404 (12.0)		419 (16.3)	207 (16.1)	212 (16.5)	
Common	15,863 (76.6)	476 (74.7)	15,387 (76.7)		2,014 (78.4)	1,003 (78.1)	1,011 (78.7)	
Bad	2,363 (11.4)	89 (14.0)	2,274 (11.3)		135 (5.3)	74 (5.8)	61 (4.8)	
Noon nap				0.10				0.11
No	14,665 (70.8)	433 (68.0)	14,232 (70.9)		1,154 (44.9)	557 (43.4)	597 (46.5)	
Yes	6,037 (29.2)	204 (32.0)	5,833 (29.1)		1,414 (55.1)	727 (56.6)	687 (53.5)	
Fruit, kg/week				0.04				0.07
<0.5	553 (2.7)	29 (4.6)	524 (2.6)		117 (4.6)	70 (5.5)	47 (3.7)	
0.5–1.5	3,747 (18.1)	116 (18.2)	3,631 (18.1)		705 (27.5)	356 (27.7)	349 (27.2)	
>3	16,402 (79.2)	492 (77.2)	15,910 (79.3)		1,746 (68.0)	858 (66.8)	888 (69.2)	
Taste				0.11				0.72
Bland	4,208 (20.3)	124 (19.5)	4,084 (20.4)		1,316 (51.2)	664 (51.7)	652 (50.8)	
Common	8,699 (42.0)	249 (39.1)	8,450 (42.1)		657 (25.6)	324 (25.2)	333 (25.9)	
Heavy	7,795 (37.7)	264 (41.4)	7,531 (37.5)		595 (23.2)	296 (23.1)	299 (23.3)	
Medication use								
Antihypertensive drugs				0.007				<0.001
No	11,166 (53.9)	310 (48.7)	10,856 (54.1)		1,354 (52.7)	583 (45.4)	771 (60.0)	
Yes	9,536 (46.1)	327 (51.3)	9,209 (45.9)		1,214 (47.3)	701 (54.6)	513 (40.0)	
Hypoglycemic drugs				0.94				<0.001
No	20,385 (98.5)	627 (98.4)	19,758 (98.5)		2,265 (88.2)	1,091 (85.0)	1,174 (91.4)	
Yes	317 (1.5)	10 (1.6)	307 (1.5)		303 (11.8)	193 (15.0)	110 (8.6)	

*Data are mean (SD) or n (%)*.

*BMI, body mass index; AST, aspartate aminotransferase; γ-GT, gamma glutamyltranspeptidase; TC, total cholesterol*.

The following result description will mainly focus on the training set.

### Performance of Data Balancing Techniques

Before data balancing techniques were applied, we observed high AUCs (mean 0.643), very high accuracy (97%), very high specificity (100%), and mean kappa value of 0.97, but very low sensitivity (0), in all four analysis methods. After including laboratory data, similar patterns were found with mean AUCs, accuracy, specificity, kappa, and sensitivity of 0.647, 97%, 100%, 0.97, and 0, respectively.

After data balancing techniques were applied, we can observe improvements in sensitivity and decreases in specificity and AUCs. Higher AUCs were obtained from the analysis method applied with RUS than SMOTE in general (mean 0.623 vs. 0.512). In addition, in RUS-applied models, higher sensitivity (mean 63.9% vs. 14.4%), higher kappa (mean 0.022 vs. −0.004), lower specificity (53.7% vs. 84.4%), and lower accuracy (54.0% vs. 83.3%) were observed compared with SMOTE-applied methods. Moreover, extreme values were found in SMOTE-applied models, but not RUS (e.g., SMOTE-applied RF has 4.80 sensitivity, 95.2% specificity, and 92.7% accuracy).

### Inclusion of Laboratory Data

Although adding the laboratory variables elevated overall model performance, no significant improvement was observed (Table 2). Reductions were only observed regarding specificity and accuracy in SMOTE-applied LR and SL and AUCs for RUS-applied XGBoost, but also with a limited range.

**Table 2:** Performance of machine learning methods in different datasets with different data balancing methods.

	**Model**	**Balancing methods**	**CSPPT**					**NCC validation**				
			**AUC**	**Sensitivity**	**Specificity**	**Accuracy**	**Kappa**	**AUC**	**Sensitivity**	**Specificity**	**Accuracy**	**Kappa**
No laboratory data	RF	Null	0.651	0	100	97	0.970	0.565	0	100	50.0	0.500
	XG		0.640	0	100	97	0.970	0.552	0	100	50.0	0.500
	LR		0.641	0	100	97	0.970	0.595	0.2	100	50.1	0.002
	SLR		0.641	0	100	97	0.970	0.610	0	100	50.0	0.500
	RF	RUS	0.629	69.4	51.7	52.2	0.025	0.562	60.2	46.1	53.2	0.063
	XG		0.624	67.2	49.0	49.5	0.018	0.580	72.6	36.1	54.3	0.086
	LR		0.626	58.6	57.9	57.9	0.022	0.582	62.4	50.2	56.3	0.125
	SLR		0.616	60.2	56.3	56.4	0.022	0.579	65.4	46.3	55.8	0.117
	RF	SMOTE	0.524	5.4	94.3	91.7	−0.002	0.507	4.6	97.0	50.8	0.016
	XG		0.514	9.7	87.4	85.1	−0.011	0.512	12.1	87.7	49.9	−0.002
	LR		0.505	21.5	78	76.3	−0.001	0.483	35.9	63.4	49.6	−0.007
	SLR		0.505	21	77.9	76.2	−0.003	0.483	36.1	63.3	49.7	−0.006
With laboratory data	RF	Null	0.654	0	100	97	0.970	0.580	0	100	50.0	0.500
	XG		0.621	0	100	97	0.970	0.576	0	100	50.0	0.500
	LR		0.656	0	100	97	0.970	0.584	0.2	100	50.1	0.002
	SLR		0.657	0	100	97	0.970	0.610	0	100	50.0	0.500
	RF	RUS	0.640	72.0	52.8	53.4	0.030	0.584	68.5	42.9	55.7	0.114
	XG		0.620	67.7	50.9	51.4	0.022	0.577	73.7	32.4	53.0	0.061
	LR		0.634	60.8	58.6	58.6	0.026	0.538	62.9	42.8	52.8	0.057
	SLR		0.639	60.2	57.4	57.5	0.023	0.579	65.4	46.3	55.8	0.117
	RF	SMOTE	0.533	4.8	95.2	92.5	0.000	0.531	3.0	97.8	50.4	0.008
	XG		0.525	10.8	88.1	85.8	−0.005	0.526	13.9	88.2	51.1	0.021
	LR		0.538	30.6	75.9	74.5	0.015	0.498	43.4	56.9	50.2	0.003
	SLR		0.538	30.6	75.9	74.5	0.015	0.497	43.0	57.4	50.2	0.004

*RF, random forest; XG, XGBoost; LR, logistic regression; SLR, stepwise logistic regression; RUS, random under-sampling; SMOTE, synthetic minority over-sampling technique; and AUC, area under the receiver operating characteristic curve*.

With data balancing techniques, the inclusion of laboratory data improved AUCs (mean 0.58 vs. 0.57), sensitivity (42.2% vs. 39.1%), specificity (69.3% vs. 69%), accuracy (68.5% vs. 68.2%), and kappa (0.016 vs. 0.008) compared with the exclusion of laboratory data.

### Performance of Analysis Methods

Overall, the RF method appeared to have the highest mean AUCs (Null + RUS + SMOTE/3) of 0.601, and SLR showed the lowest average AUCs of 0.587 before adding laboratory variables. After the inclusion of laboratory data, highest and lowest mean AUC values were found in SLR and XGBoost with values of 0.612 and 0.589, respectively.

In RUS-applied methods, both with and without laboratory data, the highest mean AUC of 0.642 was found in RF, which was also displaced with the highest average sensitivity of 70.7%. Under the same circumstances, the lowest AUC was found in XGBoost with a mean value of 0.622. The lowest sensitivity (59.7%) was observed in the LR model. A similar result was found in SMOTE-applied models as well, with RF having the highest mean AUC (0.528) and XGBoost having the lowest value (0.520) both before and after the inclusion of laboratory data.

Before adding laboratory variables, in the methods processed with both RUS and SMOTE, the RF model showed the highest mean AUC of 0.577, whereas SLR had the lowest AUC value of 0.561. In the analysis method, including laboratory variables and applying RUS and SMOTE, SLR was observed to have the highest mean AUC of 0.589, while XGBoost appeared to have the lowest AUC of 0.573.

### NCC Dataset (External Validation Set)

**Figure 2:**
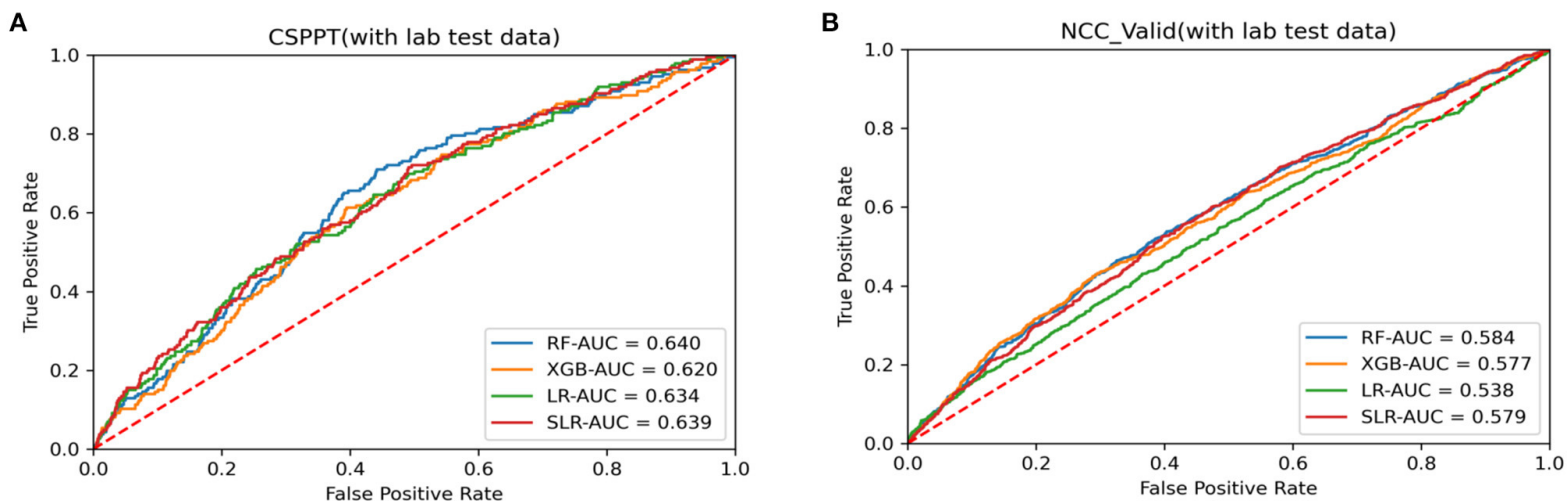
Receiver operating characteristic (ROC) curves for data analysis methods with laboratory data in **(A)** CSPPT dataset (training set) and **(B)** NCC dataset (external validation set).

In general, similar findings to that of the training set were observed in the NCC dataset as well. The best model performance was obtained in the RUS-applied RF with the highest AUC value of 0.584, which outperformed other tested methods (Table 2). Receiver operating characteristic (ROC) curves were generated to examine and compare the performance of four RUS-applied analysis methods in both the CSPPT and NCC datasets with the inclusion of laboratory data (Figure 2). Results with the exclusion of laboratory data are presented in Supplement Figure 1.

### Selection of Stroke Predictors

**Figure 3:**
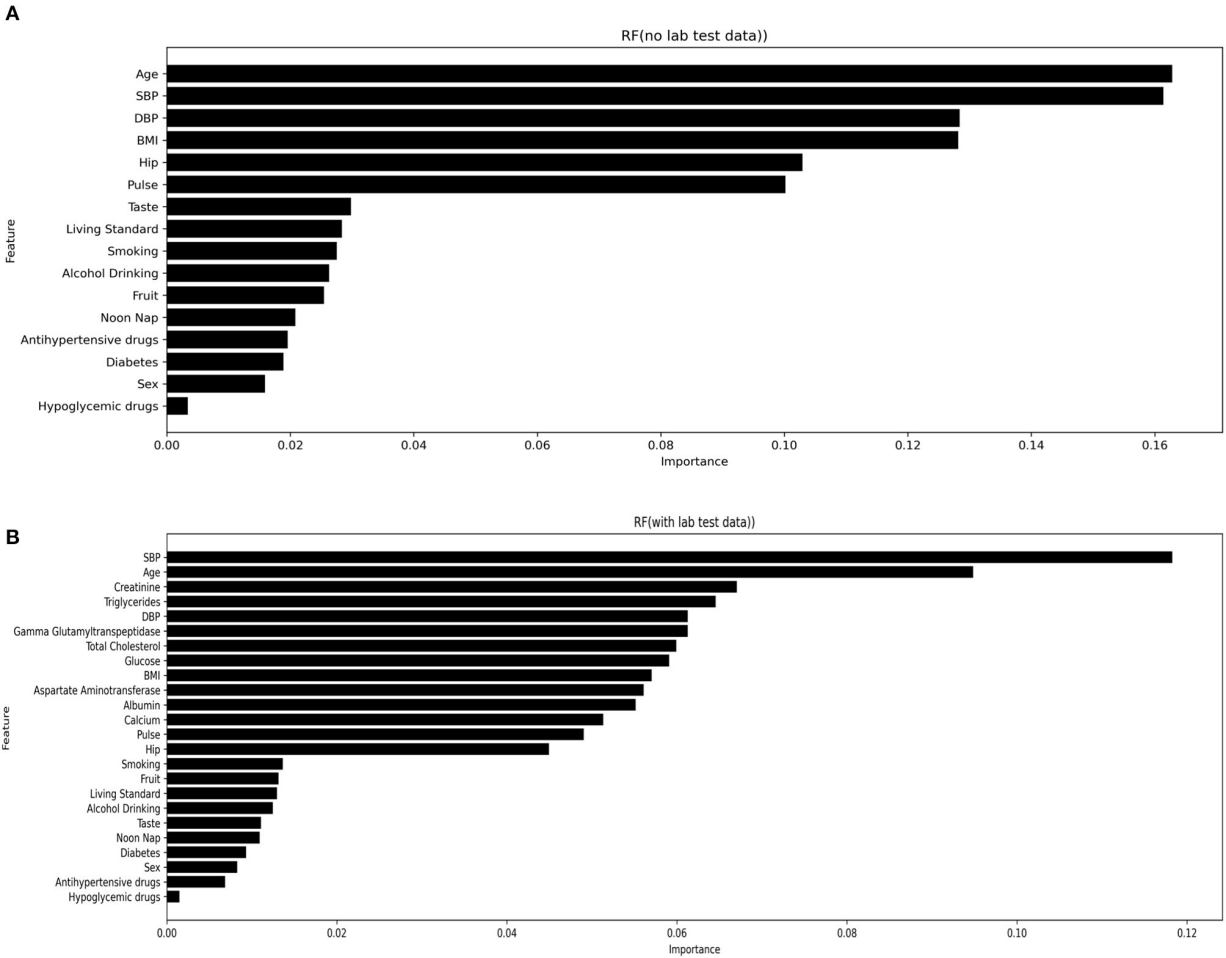
Most important variables from RUS-applied RF with both inclusion **(A)** and exclusion **(B)** of laboratory variables.

Important variables were selected according to the following techniques: Standardized regression coefficients were used to evaluate the importance of variables in the LR and SLR models; the Gini coefficient (average contribution) was calculated for each variable across all branches in the RF model; the relative numbers of times of a single variable in the full data distribution; and the Gini coefficient was identified for the XGBoost model. The top 25 variables were selected from the most optimal stroke prediction model, being RUS-applied RF with the inclusion of laboratory data, as stroke risk predictors. Figure 3 highlights the most important variables in the RUS-applied RF model with and without laboratory variables. Supplement Figure 2 presents the most important variables from RUS-applied XGBoost method with the inclusion and exclusion of laboratory variables. We can observe different orders and more diversity when more variables were added, but SBP, age, creatinine, triglycerides, and DBP were most commonly identified as the top five important variables.

## Discussion

The optimal stroke prediction model was not well established and often varies across different studies. Our study not only developed an effective stroke prediction model using machine learning analysis, but also revealed important insights into machine learning-based prediction models in general. To our knowledge, this study is by far the first and largest study that builds machine learning-based stroke prediction model using hypertensive population data.

Heo et al.'s (17) study, which focused on acute ischemic stroke, reported DNN (deep neural network) as the optimal prediction method. Wu et al.'s (18) study found that SMOTE-applied RLR outperformed other tested models in an older Chinese population for predicting the risk of stroke. Ambale-Venkatesh et al. (4) identified RF to be the most effective cardiovascular risk (including stroke) prediction model among nine tested methods in a multiracial population. Moreover, despite showing poor performance in the current study, XGBoost has been previously suggested to be the most effective prediction model for various populations and outcomes (19, 20). The current study found RUS-applied RF method with the inclusion of laboratory variables to be the most effective stroke prediction model in Chinese hypertensive adults. Ascribing to the large sample size and comprehensive study design, we believe the current study is representative in the field of machine learning-based prediction method development. Moreover, with displayed best performance in both training and validation set, the developed stroke prediction method in the present study showed robust universality and accuracy.

Analysis methods, including logistic regression and machine learning, can be disrupted by imbalanced data (21). Data balancing techniques are necessary when pre-processing an imbalanced database (22). The current study had a 1:31 stroke-to-non-stroke ratio, which indicated an imbalance. Before data balancing techniques were applied, the sensitivity of all models was 0, which suggested poor performance regardless of high AUCs. It would thus be inappropriate to directly utilize the raw data. Previous studies have demonstrated the patterns with low sensitivity in the raw model and an overall improvement after applying data balancing techniques (22, 23), which is concomitant with the current study. However, the enhancement effectiveness of applying data balancing technique varied significantly between individual models. In the current study, compared with null model, the sensitivity in the RUS-applied RF with laboratory data model increased from 0 to 72 but was only brought up to 4.8 when the same model was treated by SMOTE. In addition, different AUCs were observed when the same analysis model was applied with different data balancing techniques (18, 24).

To examine the effectiveness of the increment variables on the overall performance of the analysis model, laboratory variables included and excluded were analyzed, which is another unique feature of the present study. To our knowledge, some previous studies have included laboratory variables as a part of stroke prediction (4, 25, 26), but few studies have conducted separate analyses. As given in Table 2, when laboratory results were included, the overall performance of the analysis method was improved both before and after data balancing techniques were applied. Consistent results were reported in An Dinh et al.'s (19) study, which used machine learning methods to predict diabetes and cardiovascular disease, and all AUCs had an average increase of 0.7% after laboratory tests were included.

The targeted participants in our study consisted of population with a higher risk of stroke compared with previous studies (Table 1). Ascribing to the nature of strictly processed RCT, endpoint events were accurately collected, and all variables were presented with veracity and reliability. Compared with Gu D et al.'s study, which aimed to develop a 10-year stroke predicting equation in a Chinese population, our study had a higher baseline age (48 vs. 60), higher baseline SBP (123.6 mmHg vs. 165.8 mmHg), a more rural population, higher antihypertensive drug usage, and less current smokers (25). On the contrary, in contrast to Bharath A et al.'s study, which focused on cardiovascular event prediction by machine learning, our study solely focused on the first stroke and had more current smokers, higher usage of antihypertensive drugs, higher baseline SBP (165.8 mmHg vs. 126.6 mmHg), and lower BMI (24.8 kg/m^2^ vs. 28.34 kg/m^2^) (4).

Our study underlines the importance of validation. To demonstrate a trained model is effective, merely succeeding on the original data is not sufficient. It is essential to adduce evidence that such a developed model can perform well in other datasets. However, many review articles have pointed out the general lack of validation or insufficient validation (27, 28). Internal validation enables researchers to quantify and estimate positives from data processing, while verifying results from the training set (29). Furthermore, the trained model should be conducted in the external validation set which contains different data from the training set to examine and evaluate the developed model's performance (28). Studies that lack a thorough validation are relatively less power enough to be convincible of the developed models.

Our study findings have provided important clinical and public health implications. The selection of stroke risk predictors often differs according to various studies (4), even when targeting the same race. The current study focused on Chinese rural hypertensive adults and suggested that SBP, age, creatinine, triglycerides, and DBP are the top five stroke risk predictors. Nevertheless, the top 5 important variables from Wu's (18) study were sex, LDLC, GLU, hypertension, and UA. This difference could be caused by the fact that Yafei Wu et al. focused on an elderly population with a median age of 83 years, while our study has a mean age of 60 years. In addition, Dongfeng GU et al.'s study, with a mean baseline SBP 123.6 mmHg (SD 19.9), reported age, SBP, current smoking, diabetes mellitus, and total cholesterol as the most important variables (25). In comparison, our study has a mean baseline SBP of 165.8 mmHg (SD 18.3).

## Conclusion

Among the tested methods, the most effective stroke prediction model in Chinese rural hypertensive adults without a history of stroke is RUS-applied RF with the inclusion of laboratory variables. From the insights, the current study revealed, we provided general frameworks for building machine learning-based prediction models.

### Limitations

Some limitations are a worth concern. Our analysis was focused on a targeted population, and thus, despite the high representation (large sample size, wide age range, and higher morbidity region), further validation is needed to apply the model to larger and more diverse data. In addition, this study only used two currently popular data balancing techniques and two classic analysis methods to develop the stroke prediction models. As improvements and novel methodologies are developed in future, they should be applied and evaluated as well.

## Data Availability Statement

The original contributions presented in the study are included in the article/Supplementary Material, further inquiries can be directed to the corresponding authors.

## Ethics Statement

The studies involving human participants were reviewed and approved by the Ethics Committee of the Institute of Biomedicine, Anhui Medical University, Hefei, China. The patients/participants provided their written informed consent to participate in this study.

## Author Contributions

XX, YH, XC, and LL critically revised the protocol for research design. LL, XH, BX, RX, JuL, ZT, YS, ZZ, ZW, and YWe were responsible for implementing onsite. LL and LC performed the statistical analysis. TC and XH drafted the manuscript. YWu, JiL, YZ, XQ, XW, and HW developed the methodological approach. All authors contributed to the conception and design and approved the final version of the manuscript.

## Funding

This study was supported by funding from the following: Key R&D Projects, Jiangxi [20203BBGL73173]; the National Natural Science Foundation of China [81960074, 81730019, and 81973133]; Jiangxi Provincial Health Commission [202130440]; the National Key Research and Development Program [2016YFE0205400, 2018ZX09739010, and 2018ZX09301034003]; the Science, Technology and Innovation Committee of Shenzhen [JSGG20170412155639040, GJHS20170314114526143, and JSGG20180703155802047]; the Economic, Trade and Information Commission of Shenzhen Municipality [20170505161556110 and 20170505160926390]; and the Research Fund Program of Guangdong Provincial Key Laboratory of Renal Failure Research, Clinical Innovation Research Program of Guangzhou Regenerative Medicine and Health Guangdong Laboratory [2018GZR0201003]. The China Stroke Primary Prevention Trial (CSPPT) was jointly supported by Shenzhen AUSA Pharmed (Shenzhen, China) and national, provincial, and private funding, including from the Major State Basic Research Development Program of China (973 program; grant no. 2102 CB517703); the National Science and Technology Major Projects Specialized for Innovation and Development of Major New Drugs during the 12th Five-year Plan Period: the China Stroke Primary Prevention Trial (grant no. zx09101105), a Clinical Center grant (no. zx09401013); the National Clinical Research Center for Kidney Disease, Nanfang Hospital, Nanfang Medical University, Guangzhou, China; the State Key Laboratory for Organ Failure Research, Nanfang Hospital; and research grants from the Department of Development and Reform, Shenzhen Municipal Government (grant no. SFG 20201744). The funding organizations and/or sponsors participated in the study design, but had no role in the conduct of the study; collection, management, analysis, and interpretation of the data; preparation, review, or approval of the manuscript; or the decision to submit the manuscript for publication.

## Conflict of Interest

The authors declare that the research was conducted in the absence of any commercial or financial relationships that could be construed as a potential conflict of interest.

## Publisher's Note

All claims expressed in this article are solely those of the authors and do not necessarily represent those of their affiliated organizations, or those of the publisher, the editors and the reviewers. Any product that may be evaluated in this article, or claim that may be made by its manufacturer, is not guaranteed or endorsed by the publisher.
