# Supplementary material for: Novel Insights on Establishing Machine Learning-Based Stroke Prediction Models Among Hypertensive Adults
Source: Front Cardiovasc Med. 2022 May 6;9:901240. doi: 10.3389/fcvm.2022.901240 (PMC9120532; doi:10.3389/fcvm.2022.901240)
Supplement: Supplementary file 1 [file Data_Sheet_1.PDF]

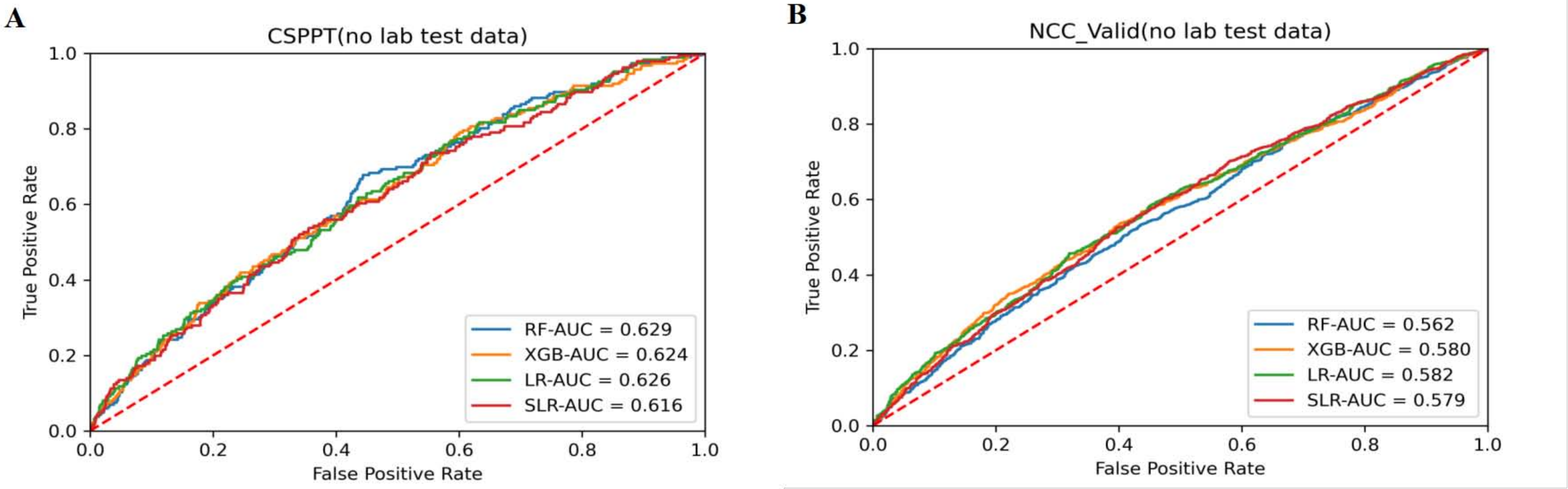

Supplement figure 1: The receiver operating characteristic (ROC) curves for machine learning methods in different datasets using RUS. Without lab test data
(A) CSPPT training dataset; (B) NCC validation dataset

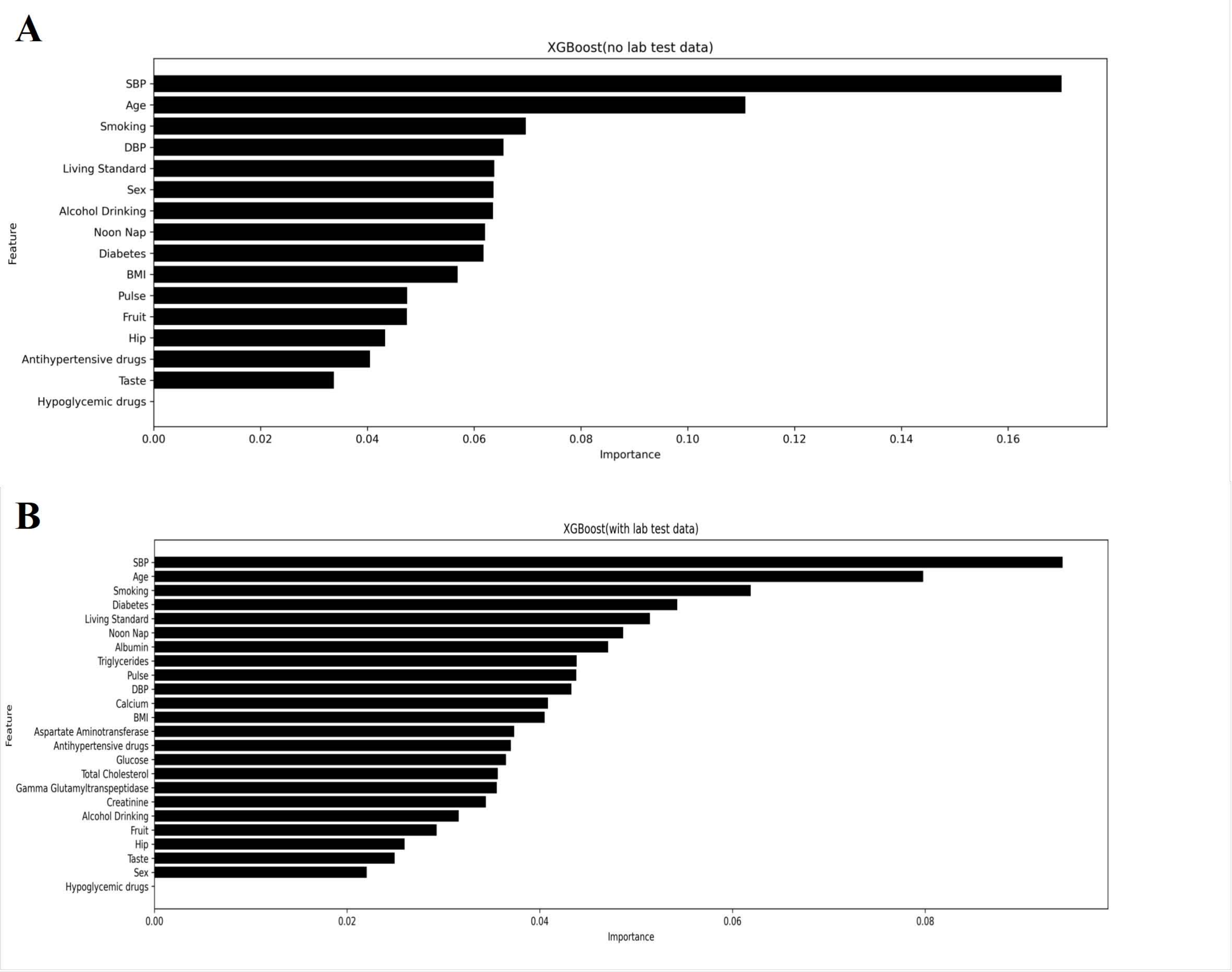

Supplement figure 2: The most important features with lab test data or without lab test data for predicting stroke using XGBoost (A)XGBoost without lab data (B)XGBoost with lab data
